# Many Genbank Entries for Complete Microbial Genomes Violate the Genbank Standard

**DOI:** 10.1002/cfg.63

**Published:** 2001-02

**Authors:** Peter D. Karp

**Affiliations:** Bioinformatics Research Group SRI International EK223, 333 Ravenswood Avenue Menlo Park CA 94025 USA

## Abstract

A survey of Genbank entries for complete microbial genomes reveals that the majority do not conform to the Genbank standard. Typical deviations from the Genbank standard include records with information in incorrect fields, addition of extraneous and confusing information within a field, and omission of useful fields. This situation results from two principal causes: genome centres do not submit Genbank records in the proper form and the Genbank, EMBL and DDBJ staffs do not enforce the database standards that they have defined.


**Comparative and Functional Genomics**
*Comp Funct Genom* 2001; **2**: 25–27.

**Conference Paper**

### Many Genbank entries for complete microbial genomes violate the Genbank standard

Peter D. Karp*
*Bioinformatics Research Group, SRI International, EK223, 333 Ravenswood Avenue, Menlo Park, CA 94025, USA*

*Correspondence to:
P. D. Karp, Bioinformatics Research Group, SRI International, EK223, 333 Ravenswood Avenue, Menlo Park, CA 94025, USA.
E-mail: pkarp@ai.sri.com*

### Abstract

**A survey of Genbank entries for complete microbial genomes reveals that the majority do not conform to the Genbank standard. Typical deviations from the Genbank standard include records with information in incorrect fields, addition of extraneous and confusing information within a field, and omission of useful fields. This situation results from two principal causes: genome centres do not submit Genbank records in the proper form and the Genbank, EMBL and DDBJ staffs do not enforce the database standards that they have defined. Copyright © 2001 John Wiley & Sons, Ltd.**

**Keywords:** **genome annotation; Genbank; bioinformatics; database standards**

### Introduction

Genome annotation is a complex process with a number of phases including gene finding, prediction of gene function, prediction of pathways and submission of the genome to the Genbank/EMBL/DDBJ databases (henceforth referred to simply as Genbank). If a submitted genome is not prepared according to the Genbank standard, the scientific community will face significant barriers in accessing and manipulating the genome annotation that was so painstakingly produced. This article presents evidence that many complete genomes within Genbank were not prepared according to the Genbank standard.

Genbank now contains 30 complete bacterial genomes. As the number of complete genomes increases, it becomes more and more important that data within Genbank are encoded in a consistent and regular form that allows computer programs to reliably extract information, since manual interpretation of those records becomes less and less feasible. For example, a computer program that attempts to search across many different Genbank entries to find a given coding region by gene name, or by gene-product name, or by the unique identifier assigned by a sequencing project, must know what Genbank feature-table qualifiers to search for each of these types of information. In isolation, none of the examples presented are that dramatic but, taken together, the scale and diversity of these malformed data creates a significant barrier to computational analysis of Genbank.

### The Genbank standard is neither followed nor enforced

The genome centres that have submitted Genbank entries for complete genomes are not following the Genbank standard (which is available at **http://www.ncbi.nlm.nih.gov/collab/FT/index.html**) and the NCBI, EMBL and DDBJ groups that accept new Genbank entries are not enforcing that standard. Figure 1 shows excerpts from three Genbank entries for complete microbial genomes or chromosomes, each of which was prepared by a different sequencing group. The left side of the figure lists the original entry; the right side of the figure shows a corrected version of the entry.

All of the entries in Figure 1 use different syntax and semantics, and all violate the Genbank standard in some way. In 1a, the product name is

Copyright © 2001 John Wiley & Sons, Ltd.

**Figure 1.** (1a–3a) Excerpts from three Genbank entries that do not conform to the Genbank standard. (1b–3b) Corrected versions of each entry that do conform to the standard

Copyright © 2001 John Wiley & Sons, Ltd.

prefixed with a variant of the gene name. In example 2a, the product qualifier simply repeats the gene name. The real product name, along with much other useful information, is buried in a text field in a form that cannot be automatically parsed by a computer program. In the case of 3a, the unique ID is in the gene qualifier and the gene name is appended to the product qualifier.

In addition, none of the entries has a label qualifier containing the unique identifier associated with each coding region. Although the specification does not require that the label qualifier be present, this unique identifier is useful for database linking.

A list of 11 non-conformant Genbank entries and a conversion of those entries to a form that does meet the standard is provided at **http://www.ai.sri.com/pkarp/misc/gbkexample.html**

### Discussion

Although it is troubling that the sequencing projects are not following the Genbank standard, it is even more troubling that the database staffs are not enforcing their own standards. An important role of the Genbank staff is ensuring that only high-quality data enter Genbank, which is the principal archive of nucleotide-sequence information for the scientific community. The Genbank staff should refuse to accept entries that do not conform to the Genbank standard. Although the staff might argue that their resources are inadequate for policing every submission to Genbank, we would argue that at least a minimal level of manual checking should be performed for entries for complete genomes. Literally 15 minutes of inspection would suffice to identify many of the problems we have listed. Inspection of every coding sequence in a file is generally not necessary, because these files are typically generated by programs that create the same non-conformant fields in a systematic fashion for every coding region.

Furthermore, some automated checks should be performed on every incoming entry, such as verifying that the contents of the EC qualifier is a valid EC number, verifying that the contents of the label qualifier are unique across the entry, and verifying that a label qualifier is provided for every coding region.

Some simple rules to remember when formulating Genbank entries are:

- Put each piece of information in the appropriate qualifier.
- Supply as many qualifiers for each coding sequence as can reasonably be provided.
- Do not attempt to be creative by adding additional information into a given qualifier. For example, adding multiple synonyms for the gene name inside a given gene qualifier violates the specification and could produce erroneous results in software that processes that qualifier.

See **http://www.ai.sri.com/pkarp/misc/gbkexample.html** for more examples of conformant Genbank entries.

### Acknowledgements

This work was sponsored by Grant 1-R01-RR07861-01 from the National Institutes of Health.

Copyright © 2001 John Wiley & Sons, Ltd. 

